# Circulating Bacterial DNA as Plasma Biomarkers for Lung Cancer Early Detection

**DOI:** 10.3390/microorganisms11030582

**Published:** 2023-02-25

**Authors:** Huifen Zhou, Jipei Liao, Qixin Leng, Molangur Chinthalapally, Pushpa Dhilipkannah, Feng Jiang

**Affiliations:** 1 Department of Pathology, University of Maryland School of Medicine, 10 South Pine Street, MSTF 7th Floor, Baltimore, MD 21201, USA; 2 Environmental Science and Technology, College of Agriculture and Natural Resources, University of Maryland, College Park, MD 20742, USA

**Keywords:** microbes, diagnosis, NSCLC, early, plasma

## Abstract

**Simple Summary:**

Pathogenic bacteria play a crucial role in tumor development. Our study analyzed the presence of bacteria related to lung cancer in lung tumors, normal lung tissues, and plasma from lung cancer patients. Three bacteria (*Selenomonas*, *Streptococcus*, and *Veillonella*) showed consistent changes in plasma and higher DNA abundances in the plasma of cancer patients compared with healthy controls. The use of these three bacteria as a plasma biomarker panel can identify lung cancer, regardless of histology and stage. Our findings were validated in additional clinical specimens, demonstrating the potential of circulating bacterial DNA as plasma biomarkers for lung cancer.

**Abstract:**

Lung cancer is a leading cause of cancer deaths and early diagnosis can significantly improve outcomes. Pathogenic bacteria have been shown to play a role in tumorigenesis and its analysis provides a new approach for cancer diagnosis. To evaluate the potential of bacteria as plasma biomarkers for early lung cancer detection, we analyzed eight lung-cancer-related bacterial genera in 58 lung cancer patients and 58 controls using ddPCR. Our results showed that five genera had higher DNA abundance in lung tumor tissues compared with normal tissues. Three of these genera (*Selenomonas*, *Streptococcus*, and *Veillonella*) displayed consistent changes in plasma, with higher DNA abundance in lung cancer patients compared with controls. When used as a panel, these three bacterial genera had a sensitivity of 75% and specificity of 78% for lung cancer detection, regardless of stage or histology. The performance of this biomarker panel was confirmed in an independent cohort of 93 lung cancer cases and 93 controls. Thus, circulating bacterial DNA has the potential to be used as plasma biomarkers for early lung cancer detection.

## 1. Introduction

Lung cancer is the leading cause of cancer deaths, with two main forms: Small cell lung cancer (SCLC) and non-small cell lung cancer (NSCLC) [1]. NSCLC makes up over 85% of lung cancer cases and is mainly composed of adenocarcinoma (AC) and squamous cell carcinoma (SCC) [1]. Early detection and effective treatment of NSCLC can significantly reduce mortality rates [1]. The need for accurate biomarkers to identify early-stage lung cancer in clinical settings is crucial.

The microbiome refers to the collective genetic material of all microorganisms, including bacteria, fungi, viruses, archaea, and protists. It has been proposed that the microbiome plays a crucial role in tumor development through various mechanisms, including disruption of local immune barriers, toxin production, genome instability, and release of tumor-promoting metabolites [2]. Bacteria have been well-studied in the context of malignancies. High levels of *Acidovorax* were found in lung squamous cell carcinoma tissues with p53 mutations [3]. Elevated DNA abundances of *Streptococcus* and *Veillonella* have been linked to dysregulated ERK and PI3K functions in lung cancer cells [4]. In a previous study, we showed that *Streptococcus* promotes lung tumorigenesis by activating NF-kB pathways through binding PspC to PAFR [5]. Our recent work showed that the analysis of *Acidovorax* and *Veillonella* in sputum could improve the detection and classification of NSCLC [6]. Thus, the microbiome may offer potential biomarkers for early detection and diagnosis of cancer [7].

Blood is a commonly used, minimally invasive body fluid for biomarker and liquid biopsy development. Despite numerous proposed tumor-specific molecular alterations, such as cell-free tumor DNA (ctDNA) or ctDNA methylation profiles, none have been widely adopted in clinical settings due to limited performance. Circulating bacterial DNA, which can be detected in plasma, has emerged as a promising biomarker for cancer diagnosis and prognosis, although its source remains unclear. For example, Xiao et al. found that plasma bacterial DNA profiles could differentiate colorectal cancer patients from both colorectal adenoma patients and healthy controls [8]. Measuring *E. coli*, *B. fragilis*, and *C. albicans* in plasma has been shown to monitor the outcomes of colorectal cancer patients [9,10]. Poore et al. developed circulating bacterial DNA signatures to detect various types of cancer, even in early-stage tumors without genomic abnormalities [11]. However, the use of circulating bacterial DNA as biomarkers for early detection of lung cancer has not been well documented. In this study, we aim to determine if bacteria can be developed as plasma biomarkers for the diagnosis of early-stage NSCLC.

## 2. Materials and Methods

### 2.1. Cases and Specimens

The protocol was approved by the local IRB/Ethics Committee. First, we obtained the paired lung tumor tissues and noncancerous lung tissues and blood of 58 NSCLC patients from the University of Maryland Medical Center (UMMC). Blood samples were also collected from 58 cancer-free smokers. We enrolled the cases and controls with matching demographic features, including age, race, gender, and smoking status et al., to reduce the confounding effects on bacterial analysis. Blood samples of the 58 NSCLC cases and 58 cancer-free subjects were used as a discovery set for identification of plasma biomarkers (Supplementary Table S1). Second, we recruited lung cancer cases and cancer-free controls and collected blood in the Lung Nodule Clinic of UMMC. Blood was processed within one hour of the collection for plasma preparation. We enrolled a total of 93 NSCLC patients and 93 cancer-free smokers. The cohort was used as a validation set for the development of biomarkers. All participants did not have active bacterial infection as confirmed through laboratory testing. Supplementary Table S2 shows characteristics of the validation cases and controls.

### 2.2. Bacteria and Culture Conditions

Bacteria, including Azoarcus oleivorans (ATCC2411), Aeromicrobium fastidiosum (ATCC12713), Acidovorax avenae (12530), Capnocytophaga canimorsus (ATCC35979), Fusobacterium nucleatum (ATCC25586), Haemophilus influenzae (ATCC51907), Neisseria meningitidis (ATCC13077), Streptococcus pneumoniae (ATCC6303), Selenomonas noxia (ATCC43541), and Veillonella parvula (ATCC 17742), were purchased from American Type Culture Collection (ATCC, Gaithersburg, MD, USA) and cultured as previously described [4,12].

### 2.3. Genomic DNA Isolation

We used QIAamp UCP Pathogen Mini Kit (QIAGEN, Düsseldorf, Germany) to prepare DNA from the specimens. The quality of DNA was determined by reading the optical density (OD). The ratio OD260/OD280 was calculated. DNA samples within the range of 1.6–2 were considered as pure. To secure the extraction and amplification processes correctly without contamination, we always included positive controls and negative controls in each experiment.

### 2.4. Droplet Digital PCR (ddPCR) Analysis of Bacterial DNA Abundances

Eight bacterial genera were previously proposed to be linked to NSCLC, including *Acidovorax*, *Capnocytophaga*, *Fusobacterium*, *Haemophilus*, *Neisseria*, *Streptococcus*, *Selenomonas*, and *Veillonella* [6,11,13,14,15,16,17]. We carried out ddPCR to quantify DNA copy number of bacteria by using a ddPCR platform and 2X ddPCR Supermix (Bio-Rad, Hercules, CA, USA) with primers (Supplementary Table S3). The ddPCR package (Bio-Rad) was used to calculate copies of DNA per µL as described in our previous study [6]. Contamination has the important impact on microbiome studies in plasma samples that usually have low endogenous biomass. To limit the contamination, we used strict decontamination strategies as previously developed [11,18,19]. In particular, we included positive and negative controls to ensure the extraction and amplification process correctly. The controls served to verify the absence of contamination in reagents, consumables, and the environment. Furthermore, *Azoarcus oleivorans* and *Aeromicrobium fastidiosum* were identified as ones of the commonly contaminant genera in molecular analysis [19]. We analyzed levels of *Azoarcus oleivorans* and *Aeromicrobium fastidiosum* in each sample. The samples that tested positive for the contamination-related genera might have been contaminated and were not included in the results of this study.

### 2.5. Statistical Analysis

Pearson’s association was applied to evaluate the relationship of DNA copies in tissue specimens with those in the paired plasma samples, and the association of the DNA copies with demographic information of patients. The normality test was used to determine if bacterial data were normally distributed. If the data did not follow a normal distribution, Spearman’s rank correlation was applied to the estimate correlation of bacterial genera in tissue and plasma specimens. To assess the clinical significance of biomarkers, we performed receiver–operator characteristic (ROC) and calculated the area under ROC (AUC) analyses. Logistic regression models with constrained parameters and the least absolute shrinkage and selection operator (LASSO) were used to optimize a biomarker panel.

## 3. Results

### 3.1. Bacteria Exhibited Different Abundances in Lung Tumor vs. Corresponing Normal Lung Tissues

**Figure 1:**
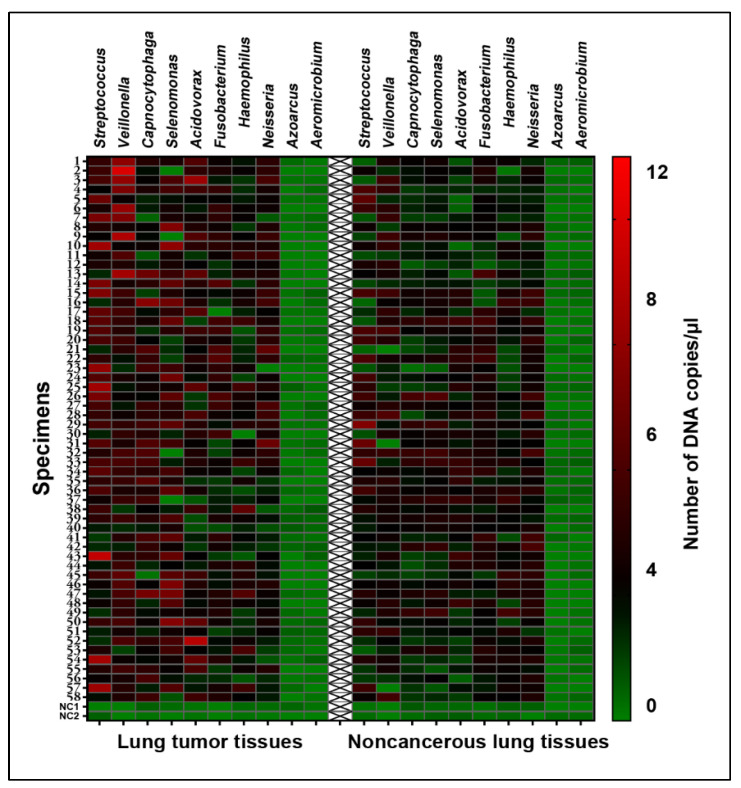
Heatmap of ddPCR analysis of DNA abundances of eight lung cancer-associated bacte-rial genera and two contaminant genera in 58 pairs of lung tumor tissues and noncancerous lung tissues, as well as negative control specimens. Columns represent DNA copy numbers/μL of bac-teria genera. The first 58 rows (1–58) represent cancer and normal lung tissues. The last two rows represent negative controls (NC 1–2) for DNA extraction and amplification, respectively. The left panel represent tumor tissues, whereas the right panel represent normal tissues.

As shown in Figure 1, negative samples included in the sample preparation and ddPCR reactions always generate negative results by ddPCR analysis. Furthermore, *Azoarcus oleivorans* and *Aeromicrobium fastidiosum*, the commonly contaminant genera, were negative in each sample (Figure 1). The results implied that no contamination was introduced in all experiments.

**Figure 2:**
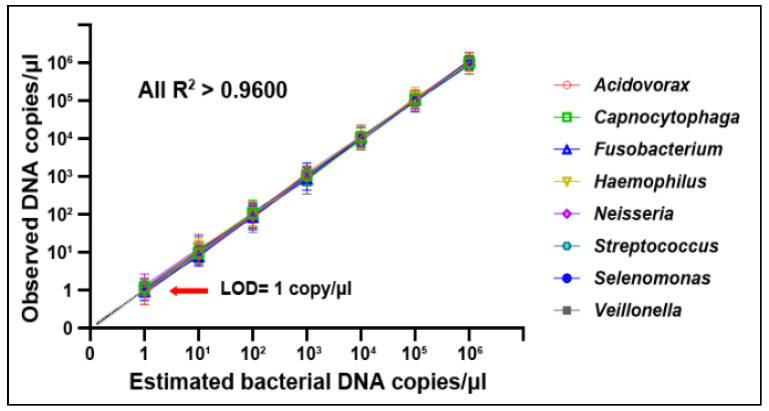
The dynamic ranges and sensitivities of ddPCR for detection of bacterial genera in serially diluted DNA standard samples. The *x*-axis represents concentrations of the dilution series, whereas *y*-axis represents measured concentrations of bacterial genera detected by ddPCR in triplets.

The serially diluted bacterial samples showed that bacteria DNA abundances detected by ddPCR were linear over the range from 1 to 10^6^ copies/μL (All R^2^  ≥  0.9600), suggesting that ddPCR had a limit of detection (LOD) of 1 copy/μL, an excellent sensitivity (Figure 2).

ddPCR with specific primers unveiled positive data for the targets and had no cross-reactivity with other bacteria (Supplementary Figure S1), indicating its high specificity. The samples were separated into three portions, and analyzed on day 1, 7 and 14. We determined inter-assay precision by measuring the coefficient of variation (CV) at different dilutions. We also determined intra-assay precision by measuring the CV over different wells. The ddPCR assay had CVs of 6.93–14.82%, demonstrating its high reproducibility and precision in the detection of bacteria (Supplementary Tables S4–S6).

Of the eight bacterial genera, five (*Acidovorax*, *Capnocytophaga*, *Streptococcus*, *Selenomonas*, and *Veillonella*) had elevated DNA abundances in the tumor tissues (Table 1) (Figure 1) (All *p* < 0.05), and the changes were not related to the histology and stage of NSCLC (All *p* > 0.05) (Supplementary Table S7).

### 3.2. Bacteria Displayed Increased Abundances in Plasma Samples of Patients Diagnosed with NSSCL

**Figure 3:**
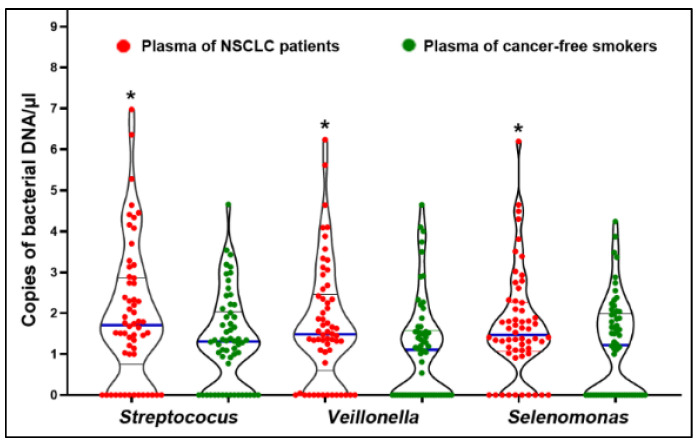
Three bacterial genera have a higher level of DNA abundances in plasma of lung cancer patients compared with controls. The solid blue line shows median. The black line shows quartiles of DNA copies of each bacterium. *, *p* < 0.05.

We used the same ddPCR technique as described above to measure bacterial DNA abundances in plasma of the 58 NSCLC patients and 58 cancer-free controls. The positive controls included in the sample preparation and ddPCR reactions always gave a response to the experiment, whereas the negative samples were correctly identified as negative. Furthermore, *Azoarcus oleivorans* and *Aeromicrobium fastidiosum* were negative in each plasma sample. Therefore, there was no contamination in the analysis of the plasma samples. Of the eight bacteria genera, *Selenomonas*, *Streptococus*, and *Veillonella* were found to have higher plasma abundance levels in cancer patients than in cancer-free smokers (*p* < 0.05) (All *p* < 0.05) (Figure 3) (Supplementary Table S8). Furthermore, the DNA abundances were independent of histology and stage of NSCLC (*p* > 0.05) (Supplementary Table S9).

**Figure 4:**
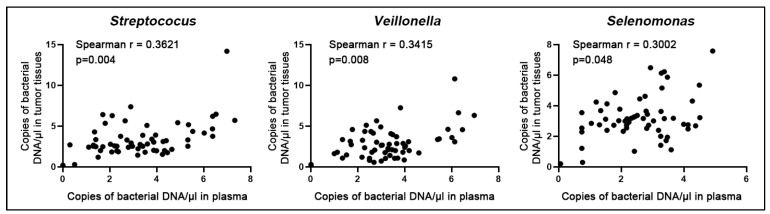
Correlation of the bacterial abundances in cancer specimens and the corresponding plasma of patients with NSCLC. Combined scatter plots show a significant correlation in DNA copy numbers of three bacterial genera (*Selenomonas*, *Streptococus*, and *Veillonella*) between lung tumor tissues and plasma samples (All *p* < 0.05; Spearman’s rank correlation).

DNA copy numbers of the three bacterial genera (*Selenomonas*, *Streptococus*, and *Veillonella*) in plasma were significantly correlated with those in the corresponding tissue specimens (Figure 4) (All *p* < 0.05; Spearman’s rank correlation). Therefore, *Selenomonas*, *Streptococus*, and *Veillonella* abundances displayed comparable levels in plasma samples vs. the matching primary cancer tissues.

### 3.3. A Panel of Three Bacterial Genera Was Developed as Plasma-Based Biomarkers for NSCLC

**Figure 5:**
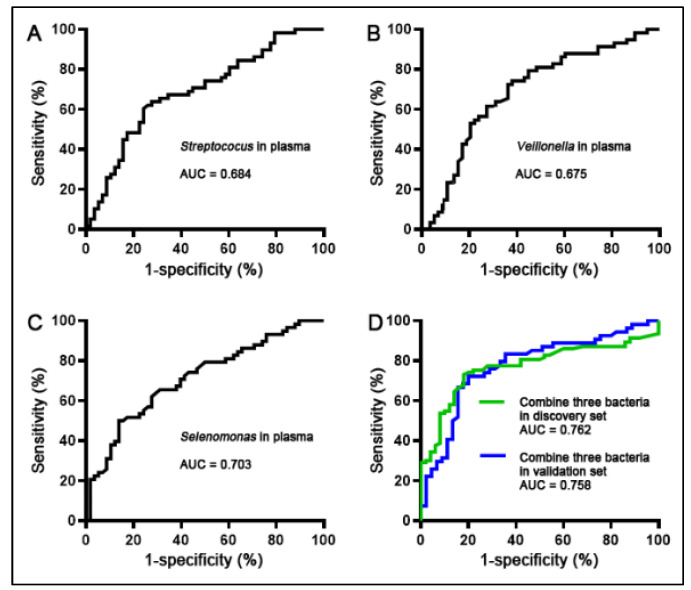
The panel of three plasma bacterial biomarkers for NSCLC detection. (**A**–**C**). The perfomanace of *Streptococus*, *Veillonella*, and *Selenomonas* was detemined by uisng AUC values. (**D**). The three plasma bacterial biomarkers used in combination in the discovery set (Green line) and the validation set (Blue line) yields 0.76 AUC, respectively, which is better than individual ones (*p*  <  0.05). However, there is no statistical difference of AUC values of the biomarker panel in discovery set vs. validation set (*p* = 0.69).

The individual bacterial genera exhibited 0.67–0.70 AUCs in differentiation of NSCLC cases from the controls, revealing 62–70% sensitivities and 60–70% specificities (Figure 5A–C) (Table 2). A logistic regression model was developed that included *Selenomonas*, *Streptococus*, and *Veillonella*. Joint examination of the three bacterial genera via the model produced an AUC of 0.76 that was significantly higher compared with those of individual ones (Figure 5D) (All *p* < 0.05) (Table 2). Consequently, the three bacterial genera used together had 75% sensitivity and 78% specificity for the detection of NSCLC (Table 2). Furthermore, correlations among abundances of the bacterial genera determined by Pearson’s correlation analysis were low (All *p* > 0.05) (Supplementary Figure S2), indicating the complementary effects of the three plasma bacterial genera. The findings further supported that the combined use of the biomarkers outclassed one alone. The plasma bacterial genera were not related to patients’ age, sex, race, and smoking history (All *p* > 0.05).

### 3.4. Confirming the Biomarkers for the Diagnostic Signifcance in Different Cases and Controls

Three bacterial genera (*Selenomonas*, *Streptococus*, *and Veillonella*) included in the biomarker panel were further tested in plasma of a different cohort comprising 93 NSCLC cases and 93 control subjects. Along with the above findings, abundances of *Selenomonas*, *Streptococus*, *and Veillonella* were higher in the plasma of lung cancer patients compared with controls (All *p* < 0.05). Moreover, the plasma biomarker panel presented comparable diagnostic performance (74% sensitivity and 76% specificity) in the validation set as did in the above discovery set (Figure 5D) (Table 2). There was no association of plasma bacterial genera with patients’ age, sex, smoking history, histology and stage NSCLC (All *p* > 0.05).

## 4. Discussion

The study shows that plasma samples contain detectable levels of circulating bacterial DNA and the elevated levels are associated with NSCLC. We identify *Selenomonas*, *Streptococus*, *and Veillonella* as a plasma biomarker panel for NSCLC that is independent of tumor histology and stage. The plasma microbiota may provide potential biomarkers for early detection of NSCLC.

Various airway microbial profiles have been observed at different airway sites of patients diagnosed with NSCLC [20]. However, the comparison of microbiome in tumor tissues and the corresponding plasma has not well been performed. In this study, we compared the DNA numbers of bacteria in cancer tissues and corresponding plasma samples of NSCLC patients. We found that changes in bacterial genera can be classified into two categories. First, bacterial genera whose changes display consistent changes in tumor tissues and plasma samples in the same manner, such as *Selenomonas*, *Streptococus*, and *Veillonella*. The aberrant bacterial abundances in plasma may reflect those in lung tumor tissues. The circulating bacterial genera in plasma could have an active rather than a passenger role in driving lung tumorigenesis, and thus have diagnostic values for lung cancer. The second category is bacterial genera (*Acidovorax* and *Capnocytophaga*) whose aberrant abundances are only observed within lung tumors. The presence of the bacterial genera in tumor tissues may play roles in the carcinogenesis of NSCLC. However, changes in the bacterial DNA copy number are not measurable in body fluids and may not provide plasma biomarkers for the disease. Further investigation of circulating bacteria is necessary in lung cancer research.

The three bacteria genera (*Selenomonas*, *Streptococcus*, and *Veillonella*) in the biomarker panel have been suggested to play a role in tumorigenesis. *Selenomonas* has been shown to be overabundant in the saliva of lung cancer patients and in tumor tissues [21]. Rodriguez et al. used exome sequencing analysis of nine cancer types and showed that tumor tissues had a higher level of *Selenomonas* abundance than did normal tissues [22]. The data generated from this study are consistent with the early reports, and further propose the potential of *Selenomonas* as a plasma biomarker for NSCLC. *Streptococcus* has been associated with various human cancers and may have a crucial role in carcinogenesis by triggering the PI3K/AKT and NF-kB pathways [4,5,23,24]. *Veillonella* has been found to be overrepresented in the lower airways of NSCLC patients and related to ERK and PI3K signaling dysregulation [4]. The current study supports the potential of *Veillonella* as a plasma biomarker for early-stage NSCLC diagnosis.

The quantification of pathogenic bacteria in plasma can be challenged by the presence of contaminating DNA [11,18,19]. To overcome this challenge, a strategy was employed in this study to reduce contamination [25]. This included the use of an optimized DNA extraction procedure, the inclusion of appropriate negative controls, the analysis of contaminating bacterial genera, and the use of ddPCR for quantification. Furthermore, we quantified bacterial genomic changes using ddPCR. The ddPCR protocol was optimized to reduce experimental variations and showed high reproducibility and precision in the quantification of bacteria. The results demonstrated the efficacy of ddPCR as a liquid biopsy for the diagnosis of lung cancer (NSCLC).

The limitations of the study include a small sample size, with only a limited number of cases and controls evaluated. To address this, a larger case–control cohort is being recruited for the prospective evaluation of the diagnostic value of the plasma biomarkers. Additionally, this study only evaluated eight lung-cancer-associated bacteria, and the sensitivity and specificity of the plasma bacterial biomarkers were not sufficient for clinical use. The number of lung-cancer-related bacteria has risen, providing additional microbiome biomarkers for the early detection and diagnosis of the malignancy [26]. We are evaluating the newly identified lung-cancer-related bacteria to develop more biomarkers to improve microbiome-based plasma tests. The use of circulating bacterial DNA as a diagnostic tool is still in its early stages, compared with the well-explored ctDNA biomarkers. We will compare the diagnostic value of circulating bacterial DNA and ctDNA biomarkers and investigate if their combination could provide better results for lung cancer diagnosis.

## 5. Conclusions

Our study demonstrates that specific bacterial genera are consistently overrepresented in both primary tumors and plasma of lung cancer patients. Additionally, we have identified bacterial biomarkers in plasma that can be used for early detection of NSCLC. These plasma biomarkers offer cost-effective diagnostic solutions and future research on the bacterial microbiome has the potential to lead to new cancer treatments.

## Figures and Tables

**Table 1:** DNA abundances of eight bacterial genera in tissues specimens of 58 lung cancer cases.

Bacterial Genera	Mean (Standard Deviation) in Lung Tumor Tissues	Mean (Standard Deviation) in Noncancerous Lung Tissues	*p* Values
*Acidovorax*	2.325 (1.376)	1.865 (0.899)	0.003780
*Capnocytophaga*	3.467 (1.187)	1.759 (0.832)	0.000382
*Fusobacterium*	3.256 (1.066)	2.355 (0.776)	0.959197
*Haemophilus*	2.075 (0.988)	1.982 (0.818)	0.309746
*Neisseria*	2.347 (1.001)	2.41 (0.8120)	0.969772
*Streptococcus*	3.245 (1.695)	2.151 (1.353)	0.000483
*Selenomonas*	2.885 (1.606)	2.115 (0.857)	0.000256
*Veillonella*	3.179 (1.544)	2.453 (1.053)	0.000042

**Table 2:** Diagnostic performance of bacterial genera in plasma.

	Sensitivity (95% CI)	Specificity (95% CI)
*Selenomonas*	70.69% (57.27% to 81.91%)	60.34% (46.64% to 72.95%)
*Streptococus*	67.24% (53.66% to 78.99%)	65.52% (51.88% to 77.51%)
*Veillonella*	62.07% (48.37% to 74.49%)	70.69% (57.27% to 81.91%)
Combined analysis of the three bacterial genera in discovery set	75.27% (65.24% to 83.63%) *	78.00% (64.04% to 88.47%) *
Combined analysis of the three bacterial genera in validation set	74.07% (60.35% to 85.04%) *	76.33% (60.46% to 87.12%) *

Abbreviations: CI, confidence interval. *, *p* < 0.05.

## Data Availability

The data that support the findings of this study are available from the corresponding author upon a reasonable request.

## Supplementary Materials

The following supporting information can be downloaded at: https://www.mdpi.com/article/10.3390/microorganisms11030582/s1, Supplementary Figure S1. The specificity of ddPCR with specific primers for detection of individual bacterial genera. Supplementary Figure S2. Correlations among three bacterial genera (*Selenomonas*, *Streptococus*, and *Veillonella*). Supplementary Table S1. Information of a discovery set comprising 58 NSCLC patients and 58 cancer-free smokers. Supplementary Table S2. Characteristics of NSCLC patients and cancer-free smokers from whom plasma samples were collected. Supplementary Table S3. Sequences of PCR primers to amplify DNA of bacterial genera. Supplementary Table S4. Day to day reproducibility of ddPCR for detection of bacterial DNA. Supplementary Table S5. Inter-assay precision of ddPCR for detection of bacterial DNA. Supplementary Table S6. Intra-assay precision of ddPCR for the detection of bacterial DNA. Supplementary Table S7. The association of abundances of bacterial genera in tissues with the age, gender, ethnic group, and tumor stage, and smoking status of the patients determined by Pearson’s correlation coefficient test. A *p*-value < 0.05 is statistically significant. Supplementary Table S8. DNA abundances of eight bacterial genera in the plasma of 58 lung cancer patients and 58 cancer-free smokers. Supplementary Table S9. The association of abundances of bacterial genera in plasma of the discovery set with the age, gender, ethnic group, and tumor stage, and smoking status of the patients determined by Pearson’s correlation coefficient test. A *p*-value < 0.05 is statistically significant.
