# Supplementary material for: Circulating Bacterial DNA as Plasma Biomarkers for Lung Cancer Early Detection
Source: Microorganisms. 2023 Feb 25;11(3):582. doi: 10.3390/microorganisms11030582 (PMC10058358; doi:10.3390/microorganisms11030582)
Supplement: Supplementary file 1 [file microorganisms-11-00582-s001.zip › microorganisms-2186422-supplementary.pdf]

Supplementary files

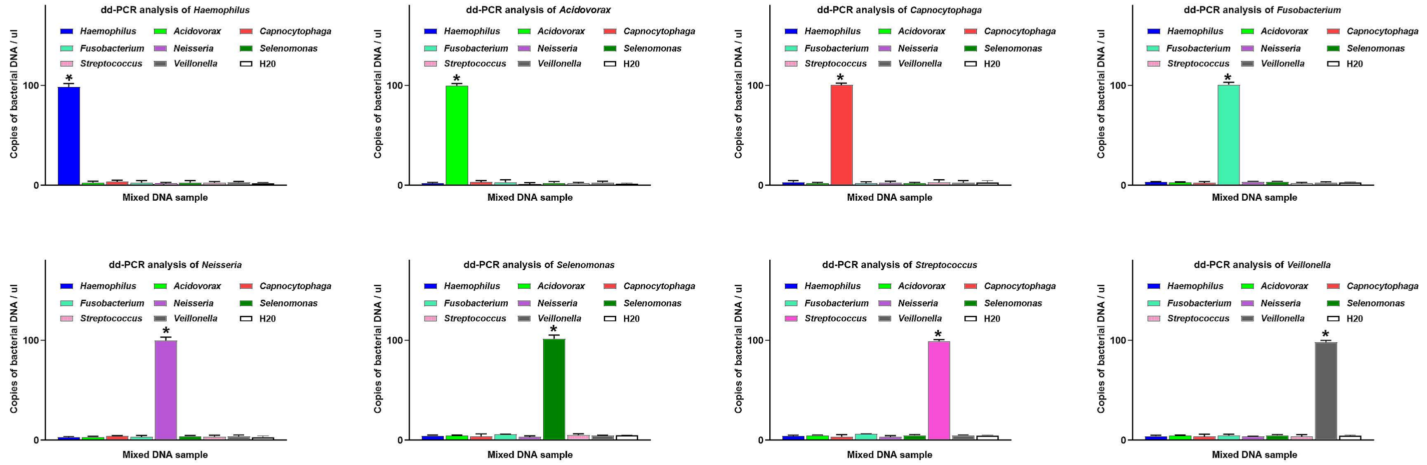

Supplementary Figure S1. The specificity of ddPCR with specific primers for detection of individual bacterial genera. The X-axis shows mixed DNA of eight bacteria genera with a final concentration of 100 copies/μL for each. The Y-axis indicates copy number/μL measured by ddPCR. Error bars represent the standard deviation from the mean of copy number generated from three replicates per sample. *, P < 0.0001.

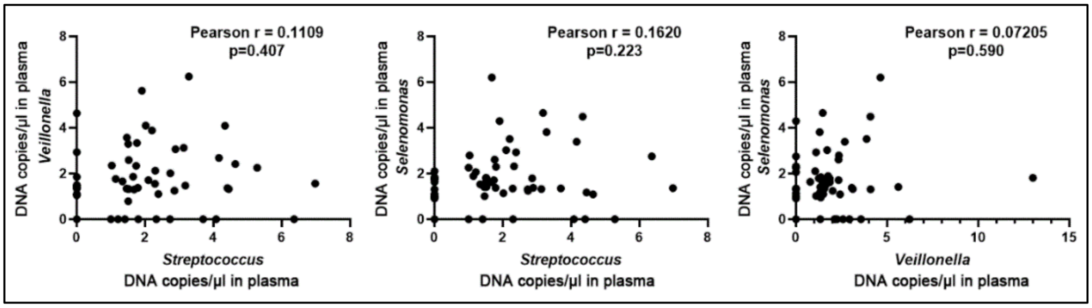

Supplementary Figure S2. Correlations among three bacterial genera (*Selenomonas*, *Streptococus*, and *Veillonella*). All correlations were calculated using Spearman rank correlation procedure. $p \leq 0.05$ as considered significant.

Supplementary Table S1. Information of a discovery set comprising 58 NSCLC patients and 58 cancer-free smokers

	NSCLC cases (n = 58)	Controls (n = 58)	P-value
Age	66.49 (SD 11.35)	64.84 (SD 10.12)	0.27
Sex			0.31
Female	21	21	
Male	37	37	
Smoking pack-years (median)	39.4	35.8	0.36
Stage			
Stage I	35		
Stage II	17		
Stage III	6		
Histological type	0		
Adenocarcinoma	37		
Squamous cell carcinoma	21		

Abbreviations: NSCLC, non-small cell lung cancer. SD, standard deviation.

Supplementary Table S2. Characteristics of NSCLC patients and cancer-free smokers from whom plasma samples were collected

	NSCLC cases (n = 93)	Controls (n = 93)	P-value
Age	66.48 (SD 10.35)	65.29 (SD 10.61)	0.27
Sex			0.31
Female	33	33	
Male	60	60	
Smoking pack-years (median)	33.2	31.6	0.17
Stage	0		
Stage I	37		
Stage II	29		
Stage III	18		
Stage IV	9		
Histological type	0		
Adenocarcinoma	60		
Squamous cell carcinoma	33		

Abbreviations: NSCLC, non-small cell lung cancer. SD, standard deviation.

Supplementary Table S3. Sequences of PCR primers to amplify DNA of bacterial genera

Genus	Forward(5′-3′)	Reverse(5′-3′)
Veillonella	CGGGTGAGTAACGCGTAATCA	CCAACTAGCTGATGGGACGC
Streptococcus	ACGCAATCTAGCAGATGAAGCA	TCGTGCGTTTTAATTCCAGC
Selenomonas	ACRCGTAGRCAACCTGCCG	CGATCCGAAGACCTTCTTCAC
Neisseria	CTGTTGGGCARCWTGAYTGC	GATCGGTTTTRTGAGATTGG
Fusobacterium	CAAGCGGTGGAGCATGTG	CTAAGATGTCAAACGCTGGTAAGG
Capnocytophaga	TGGWCAATGGTCGGAAGACTG	CCGCTACACTACACATTCCA
Acidovorax	GTCATCCTCCACCAACCAATAC	GTCTATACCGGACCAACAACAA
Haemophilus	AGCGGCTTGTAGTTCCTCTAACA	CAACAGAGTATCCGCCAAAAGTT

Supplementary Table S4. Day to day reproducibility of ddPCR for detection of bacterial DNA

Analyzed samples	CV (%)
Acidovorax	7.883
Capnocytophaga	8.134
Fusobacterium	11.982
Haemophilus	10.740
Neisseria	11.558
Streptococcus	11.809
Selenomonas	6.936
Veillonella	8.786

Supplementary Table S5. Inter-assay precision of ddPCR for detection of bacterial DNA

Analyzed samples	CV (%)
Acidovorax	7.826
Capnocytophaga	12.960
Fusobacterium	10.859
Haemophilus	14.820
Neisseria	12.038
Streptococcus	7.830
Selenomonas	6.938
Veillonella	7.112

Supplementary Table S6. Intra-assay precision of ddPCR for detection of bacterial DNA

Analyzed samples	CV (%)
Acidovorax	10.642
Capnocytophaga	8.139
Fusobacterium	8.313
Haemophilus	7.718
Neisseria	10.119
Streptococcus	10.293
Selenomonas	6.848
Veillonella	14.028

Supplementary Table S7. The association of abundances of bacterial genera in tissues with the age, gender, ethnic group, and tumor stage, and smoking status of the patients determined by Pearson's correlation coefficient test. A p-value < 0.05 is statistically significant.

	Age	Gender	Race	Smoking status	Stage	Histology
Genera	Correlation coefficients (P-value)	Correlation coefficients (P-value)	Correlation coefficients (P-value)	Correlation coefficients (P-value)	Correlation coefficients (P-value)	Correlation coefficients (P-value)
Acidovorax	-1.342 (P=0.376)	-0.175 (P=0.553)	-0.423 (P=0.345)	-0.743 (P=0.332)	-0.932 (P=0.365)	-0.234 (P=0.543)
Capnocytophaga	-0.471 (P=0.275)	0.254 (P=0.532)	-0.345 (P=0.566)	-0.445 (P=0.634)	-0.343 (P=0.267)	0.532 (P=0.343)
Fusobacterium	-0.634 (P=0.785)	-0.8756 (P=0.543)	0.546 (P=0.254)	-0.107 (P=0.288)	0.235 (P=0.671)	-0.754 (P=0.345)
Haemophilus	0.567 (P=0.345)	0.175 (P=0.239)	-0.353 (P=0.641)	-0.476 (P=0.518)	-0.367 (P=0.439)	-0.876 (P=0.244)
Neisseria	-0.865 (P=0.643)	-0.253 (P=0.567)	-0.497 (P=0.764)	0.456 (P=0.342)	-0.246 (P=0.524)	0.643 (P=0.235)
Streptococcus	-0.523 (P=0.743)	-0.454 (P=0.767)	-0.664 (P=0.868)	-0.643 (P=0.631)	-0.743 (P=0.746)	-0.865 (P=0.543)
Selenomonas	-0.359 (P=0.364)	-0.853 (P=0.245)	-0.328 (P=0.767)	-0.654 (P=0.543)	-0.752 (P=0.976)	-0.864 (P=0.234)
Veillonella	-0.676 (P=0.124)	-0.975 (P=0.244)	-0.773 (P=0.235)	-0.123 (P=0.754)	0.975 (P=0.345)	-0.524 (P=0.865

Supplementary Table S8. DNA abundances of eight bacterial genera in plasma of 58 lung cancer patients and 58 cancer-free smokers

Bacterial genera	Mean (SD) in plasma of lung cancer patients	Mean (SD) in plasma of cancer-free smokers	p values
Acidovorax	0.065 (0.030)	0.073 (0.020)	0.982583
Capnocytophaga	0.046 (0.028)	0.039 (0.023)	0.672843
Fusobacterium	0.065 (0.051)	0.061 (0.050)	0.586546
Haemophilus	0.045 (0.028)	0.042 (0.032)	0.942346
Neisseria	0.070 (0.064)	0.068 (0.059)	0.765565
Streptococcus	1.718 (1.068)	1.310 (1.012)	**0.021330**
Selenomonas	1.479(1.029)	1.221 (1.017)	**0.022373**
Veillonella	1.486 (1.046)	1.105 (1.023)	**0.015707**

SD: standard deviation.

Supplementary Table S9. The association of abundances of bacterial genera in plasma of the discovery set with the age, gender, ethnic group, and tumor stage, and smoking status of the patients determined by Pearson's correlation coefficient test. A p-value < 0.05 is statistically significant.

	Age	Gender	Race	Smoking status	Stage	Histology
Genera	Correlation coefficients (P-value)	Correlation coefficients (P-value)	Correlation coefficients (P-value)	Correlation coefficients (P-value)	Correlation coefficients (P-value)	Correlation coefficients (P-value)
Acidovorax	0.164 (P=0.754)	-0.429 (P=0.633)	-0.421 (P=0.754)	-0.404 (P=0.963)	-0.426 (P=0.353)	-0.196 (P=0.243)
Capnocytophaga	0.298 (P=0.565)	-0.187 (P=0.453)	0.303 (P=0.455)	0.877 (P=0.574)	-0.327 (P=0.435)	-0.771 (P=0.435)
Fusobacterium	-0.257 (P=0.556)	0.653 (P=0.345)	-0.315 (P=0.474)	-0.658 (P=0.324)	0.249 (P=0.434)	0.819 (P=0.864)
Haemophilus	0.728 (P=0.464)	-0.106 (P=0.235)	0.567 (P=0.538)	0.389 (P=0.234)	-0.619 (P=0.453)	-0.623 (P=0.236)
Neisseria	-0.548 (P=0.756)	0.772 (P=0.545)	-0.945 (P=0.297)	-0.759 (P=0.657)	0.567 (P=0.676)	0.559 (P=0.564)
Streptococcus	-0.552 (P=0.756)	0.768 (P=0.532)	-0.949 (P=0.297)	-0.763 (P=0.656)	0.563 (P=0.675)	0.555 (P=0.365)
Selenomonas	-0.565 (P=0.783)	0.754 (P=0.554)	-0.962 (P=0.754)	-0.776 (P=0.124)	0.549 (P=0.453)	0.541 (P=0.765)
Veillonella	-0.557- (P=0.776)	0.763 (P=0.545)	-0.955 (P=0.222)	-0.762 (P=0.675)	0.559 (P=0.463)	0.551 (P=0.358)
